# Radiological and CT findings in a extensive upper-limb involvement of Gorham's Disease: a case report

**DOI:** 10.4076/1757-1626-2-7499

**Published:** 2009-06-10

**Authors:** Long Xin, Zheng Tian, Shunwu Fan

**Affiliations:** 1 Orthopaedic Laboratory, Clinic Medical Research Institution, Sir Run Run Shaw Hospital, Zhejiang University School of MedicineNO.3 Qingchun Road, Hangzhou, 310016, Zhejiang ProvinceChina; 2 Department of Orthopaedics, The First Affiliated Hospital, Xinjiang Medical UniversityXinjiang Province, 830002China

## Abstract

**Introduction:**

The Gorham-Stout Syndrome (Gorham's massive osteolysis) is a rare condition in which spontaneous, progressive resorption of bone occurs. The etiology is poorly understood.

**Case presentation:**

We reported here a patient who had begun insidiously and was characterized by extensive osteolysis in the left upper limb, with progressive carpal bone resorption. The characteristic radiological and CT findings were presented and the clinical presentation, diagnosis and surgical treatment were discussed in the case.

**Conclusion:**

Gorham's disease is rarely involved in the whole upper limb. Histopathological evidences suggest that extensive osteolysis is caused by hemangiomatosis. The characteristic imaging findings may help us to differentiate from malignant neoplasm with progressive bony destruction.

## Introduction

Gorham's syndrome is a rare disorder of unknown etiology characterized by a non-malignant proliferation of vascular structures originating in bone with progressive bony destruction and often extending into surrounding soft tissues [1,2]. Any part of the skeletal system can be affected. Complications depend on the site of bony involvement, ranging from mild discomfort to death. We presented a woman who revealed extensive osteolysis due to a progressive hemangiomatosis in the left upper limb.

## Case presentation

A previously healthy 45-year-old woman was admitted with rapidly progressive pain in her left upper limb within one week, especially obvious swollen in the forearm. The pain was characterized as a dull ingravescence ache after onset despite conservative therapy. The deformity of left upper limb had strongly affected on her daily life. There was no history of trauma. Constitutional symptoms such as fever, anorexia, or weight loss were absent. Other skeletal examinations were normal.

On physical examination, multiple ill-defined swellings were palpated over the left upper limb. Some ecchymotic patches were distributed overlying the swelling. Range of motion of the left wrist was limited. No axillary lymph nodes were palpable. Laboratory investigations were normal apart from a mild elevation of the alkaline phosphatase.

**Figure 1.**
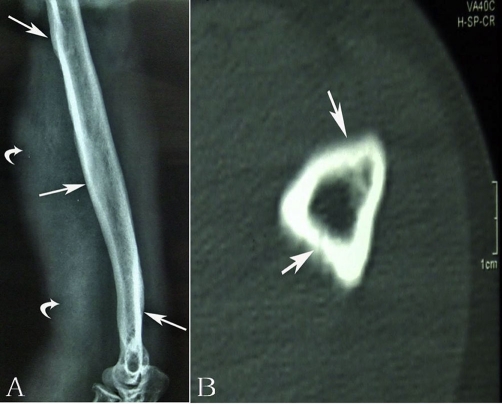
**(A)** Radiograph of left humerus revealed extensive cortical thickening and prominent sclerosis (*long arrow*) were presented along the humeral shaft. Swellon soft-tissue mass (*curved arrow*) was also visible. **(B)** CT image demonstrated extensive periosteal reaction and cortical osteolysis were presented along the proximal humerus. The medial aspect of humerus showed irregular osteolysis with pathologic fractures. (*white arrow*).

**Figure 2.**
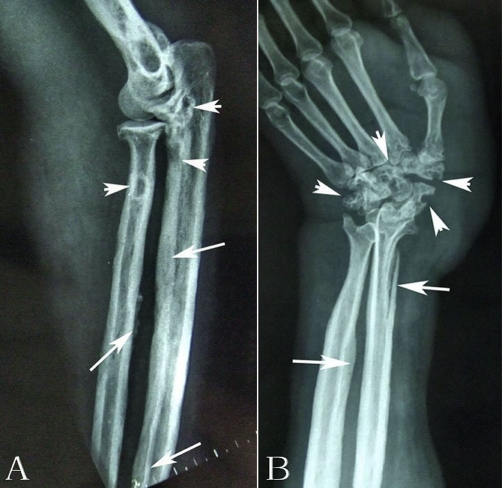
**(A)** Radiograph of forearm revealed extensive lytic defects (*head arrow*) along the proximal radius and ulna. Cortical thickening, sclerosis and aggressive periosteal reaction (*long arrows*) were presented in the proximal diaphyseal regions of both radius and ulna. **(B)** Multiple sharp margin of lytic lesions were scattered in the scaphoid, trapezium and capitate bone. (*head arrows*). Swollen soft tissue mass was also presented.

**Figure 3.**
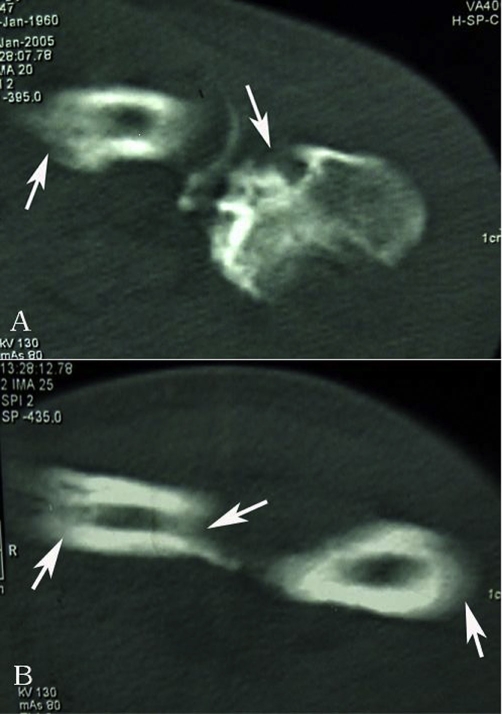
**(A)** CT image demonstrated aggressive periosteal reaction and multiple lytic lesions (*long arrows*) were presented at diaphysis of left ulna and radius. **(B)** Cortical resorption and lytic destruction (*long arrows*) was obviously noted.

**Figure 4.**
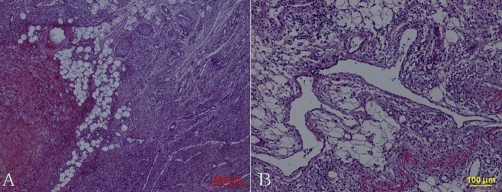
**(A)** Histologic photomicrograph (hematoxylin and eosin, ×40) showed that variable-sized ectatic vessels were interlaced in the fibrous tissue and abnormal muscle fascicles. **(B)** The dilated vessels were embedded in connective tissue. (HE, ×100).

Radiological examination revealed expansile cortical thickening and sclerosis were apparently presented along the humerus shaft (Figuer 1A). On computed tomography (CT), prominent periosteal reaction with intramedullary cortical destruction was correlated with the radiographic findings. Pathologic fractures were presented at the medial aspect of left humerus (Figure 1B). Radiograph of forearm showed expansive cortical thickening and aggressive periosteal reaction along aspect of the ulna and radius. Extensive osteolytic destructions were involved in the elbow joint. The lytic lesion of proximal radius was surrounded by a thin rim of sclerotic bone. The intramedullary and subcortical radiolucent foci resembled “patchy osteoporosis” (Figure 2A). Lamellar periosteal reaction was presented at the distal radius. The left carpal bone prominently showed multiple sharp margins of lytic lesions. The scaphoid, capitate and trapezium bones were involved. Soft tissue swellings over the abnormal bone were easily visible. (Figure 2B). CT image demonstrated that aggressive periosteal reaction and multiple lytic lesions were presented at the proximal diaphysis of left ulna and radius. Cortical resorption and lytic destruction was obviously noted. (Figure 3A & B). Because the lesion had an aggressive appearance on the plain radiographs and CT, a biopsy was performed to confirm the diagnosis. Histological examination of the specimen showed abundant intermediate-to-large ectatic vessels within a background of fibrous connective tissue intermixed with abnormal muscle fascicles. The marked proliferation of thin-walled capillaries was observed in the lesions (Figure 4A). The dilated vessels were embedded in connective tissue and small capillary-like vessels were dispersedly distributed in the stroma (Figure 4B). Pathologic examination revealed capillary hemangioma, and on the basis of the radiologic and clinical features, Gorham's disease was diagnosed. Massive resection and radiation therapy (total dose 3060 cGy) was planned. Two years after operation, there was no evidence of tumor recurrence.

## Discussion

Gorham's disease is a very rare disorder characterized by bone loss (osteolysis) often associated with uncontrolled, destructive proliferation of vascular or lymphatic capillaries within bone and surrounding soft tissue [1,3]. Most cases occur in children and young adults (usually less than 40 years of age) and no definite inheritance pattern has been reported. The natural history of Gorham's disease is unpredictable. It may spontaneously arrest or progress relentlessly until all osseous tissue disappears. Bone loss can occur in just one bone or spread to soft tissue and adjacent bones. In the case, our patient complained of dull aching pain and insidious onset of progressive weakness. The progressive bony destruction of left upper limb occurred over a period of eight months. Typical characteristics of acute spontaneous absorption of bone were noted.

Diagnosis of Gorham's disease is based on clinical and radiological features of loss of bones with histological evidence of angiomatous tumor. In most cases laboratory tests are usually within normal limits. The clinical presentation is variable, largely depending on the site of skeletal involvement. The characteristic radiographic and histopathological findings are helpful for making an early accurate diagnosis. The bones most commonly affected are the clavicle, scapula, humerus, ribs, and pelvis [4]. Radiographically, the destructive changes seen in the left upper limb mimicked malignant neoplasm due to aggressive osteolytic destruction with ill-defined lesion margins in this case. The resorption of left carpal bone was a distinguishing feature that could be differentiated from malignant neoplasm. CT scans showed extensive progressive osteolysis was involved in the whole left forearm. Soft tissue involvement was limited to the region around the bony abnormality. There does not appear to be a male or female predominance in patients with Gorham's disease. Most patients have been less than 40 years of age at the time of diagnosis [5]. The diagnosis should be made only after carefully excluding the complicated cause of osteolysis. Different forms of osteolysis such as, skeletal angiosarcoma, essential osteolysis and hereditary osteolysis must be ruled out by radiographic studies and appropriate blood tests [6]. A clinical suspicious case must be performed biopsy of the lesion.

The etiology of Gorham's disease is still speculative. There is substantial documentation that the disease has vascular or lymphatic proliferation with or without fibrosis [2,7]. Hemangiomatosis has been considered to be the characteristic feature related to the pathogenesis [1]. The progressive hemangiomatosis or lymphangiomatosis may extend to adjacent soft tissue or organs, such as the scapula, ribs or thoracic vertebra. A high morbidity and mortality is seen in patients with spinal or visceral involvement. The mechanism of bone resorption is unclear. There was no evidence of a malignant, neuropathic, or visceral component involved in our case. A biopsy confirmed extensive osteolysis was caused by progressive hemangiomatosis.

Several therapeutic modalities include radiation therapy, anti-osteoclastic medications (bisphosphonates) and alpha-2b interferon have been used in the management of Gorham's disease [8-10]. No single treatment modality has proven effective in arresting the disease. Surgical treatment options include resection of the lesion and reconstruction using bone grafts or prostheses [11,12]. In recent years, most patients have been treated with surgery or radiation therapy. Massive resection and radiation therapy was treated in our patient. Two years after surgery, there was no evidence of tumor recurrence.

## Conclusion

Gorham's disease is a very rare disorder characterized by bone loss. Our histopathological study provided good evidence that progressive osteolysis was always caused by significant vascular proliferation. Further studies are needed to elucidate the mechanisms underlying the progressive osteolysis that characterizes the disease.
